# Role of Specific B-Cell Receptor Antigens in Lymphomagenesis

**DOI:** 10.3389/fonc.2020.604685

**Published:** 2020-12-09

**Authors:** Lorenz Thurner, Sylvia Hartmann, Frank Neumann, Markus Hoth, Stephan Stilgenbauer, Ralf Küppers, Klaus-Dieter Preuss, Moritz Bewarder

**Affiliations:** ^1^ Department of Internal Medicine I, José Carreras Center for Immuno- and Gene Therapy, Saarland University Medical School, Homburg, Germany; ^2^ Dr. Senckenberg Institute of Pathology, Goethe University, Frankfurt a. Main, Germany; ^3^ Department of Biophysics, Center for Integrative Physiology and Molecular Medicine, School of Medicine, Saarland University, Homburg, Germany; ^4^ Medical School, Institute of Cell Biology (Cancer Research), University of Duisburg-Essen, Essen, Germany; ^5^ Deutsches Konsortium für translationale Krebsforschung (DKTK), Partner Site Essen, Essen, Germany

**Keywords:** B-cell receptor, antigen, lymphoma, autoreactivity, posttransnational modification, antigens of infectious origin

## Abstract

The B-cell receptor (BCR) signaling pathway is a crucial pathway of B cells, both for their survival and for antigen-mediated activation, proliferation and differentiation. Its activation is also critical for the genesis of many lymphoma types. BCR-mediated lymphoma proliferation may be caused by activating BCR-pathway mutations and/or by active or tonic stimulation of the BCR. BCRs of lymphomas have frequently been described as polyreactive. In this review, the role of specific target antigens of the BCRs of lymphomas is highlighted. These antigens have been found to be restricted to specific lymphoma entities. The antigens can be of infectious origin, such as *H. pylori* in gastric MALT lymphoma or RpoC of *M. catarrhalis* in nodular lymphocyte predominant Hodgkin lymphoma, or they are autoantigens. Examples of such autoantigens are the BCR itself in chronic lymphocytic leukemia, LRPAP1 in mantle cell lymphoma, hyper-N-glycosylated SAMD14/neurabin-I in primary central nervous system lymphoma, hypo-phosphorylated ARS2 in diffuse large B-cell lymphoma, and hyper-phosphorylated SLP2, sumoylated HSP90 or saposin C in plasma cell dyscrasia. Notably, atypical posttranslational modifications are often responsible for the immunogenicity of many autoantigens. Possible therapeutic approaches evolving from these specific antigens are discussed.

## B-Cell Development and Differentiation in the Context of Lymphoma Genesis and Autoreactivity

### B Cell Development and Generation of B-Cell-Receptor Diversity

B-lymphocytes are part of the adaptive immune system. Their main function is the production of antigen-specific antibodies during humoral immune responses. They also function as antigen presenting cells (APC) for T helper cells and can have regulatory tasks. In the course of immune responses, activated B cells can differentiate into memory B cells or antibody-secreting plasma cells. B cell development is initiated when hematopoietic stem cell-derived common lymphoid progenitors in the bone marrow differentiate into pro-B cells. Here, mediated by the lymphocyte-specific recombinases RAG1 and RAG2, and other DNA-modifying enzymes such as KU70/KU80 (1) and artemis (2), a V(D)J gene recombination of individual immunoglobulin (Ig) variable (V), diversity (D), and joining (J) genes is initiated (3). First, the Ig heavy chain is assembled through a D_H_ to J_H_ recombination, followed by a V_H_ to D_H_J_H_ joining. Multiple genes of each of the three types of genes are available for recombination, causing combinatorial diversity. As further diversification mechanisms, individual nucleotides can be deleted from the joining ends of the rearranging genes, or non-germline-encoded nucleotides (N nucleotides) are randomly inserted between the V_H_, D_H_, and J_H_ genes by terminal deoxynucleotidyltransferase (TdT) (4, 5). These processes of combinatorial and junctional diversity represent key mechanisms enabling a large variety of possible B cell receptor (BCR) reactivities given a relatively limited number of genes for immunoglobulins. After V_H_ to D_H_J_H_ joining, the heavy chain rearrangement is expressed as a pre-BCR with a non-rearranged surrogate light chain, and tested for functionality (this is needed, as about two thirds of rearrangements are out-of-frame and hence unproductive). If the first rearrangement is non-productive, a further attempt is made on the second heavy chain allele. Pre-B cells with a completed heavy chain rearrangement then perform Ig light chain gene rearrangements, beginning at the Igκ locus. The same diversification mechanisms as described for the heavy chain take place, with the exception that the light chains lack a D gene, so that V_L_ genes are directly joined to J_L_ genes. If the first rearranged VκJκ light-chain gene is not functional, further rearrangements can occur on the same allele, or on the second Igκ locus. If all these attempts fail, rearrangements of the Igλ locus occur. The combination of a rearranged heavy chain with a rearranged light chain (Igκ or Igλ) represents a further mechanism of combinatorial BCR diversity. After successful light chain rearrangement the differentiation stage of immature B cell is reached and the BCR is expressed as an IgM surface receptor. These cells are then selected against autoreactivity of the BCR (further discussed below), and the cells surviving this selection process exit the bone marrow and become mature, naive B cells, co-expressing the BCR as IgM and IgD molecules, mediated by differential splicing of the IgH transcripts.

### Oncogenic Translocations During the V(D)J Rearrangement

The rearrangement processes of Ig heavy and light chain genes, which are accompanied by DNA double strand breaks, bear the inherent risk of causing oncogenic chromosomal translocations of protooncogenes, which bring the translocated oncogenes under control of the Ig enhancers. As these are highly active in B cells, this causes a constitutive, deregulated expression of the oncogenes. These translocations are often characteristic for certain B-cell Non-Hodgkin’s lymphoma entities: In mantle cell lymphoma (MCL) the gene encoding cyclin D1 (*CCND1*) is characteristically translocated into the IgH locus (t(11;14) (q13;q32)) and in follicular lymphoma (FL) *BCL2*-IgH translocations (t(14;18)(q31;q21)) are seen in more than 90% of cases (6). Despite these translocations, a functional BCR is regularly still expressed by the respective lymphomas, strongly indicating that the cells, despite carrying these oncogenic translocations, still depend on expression of a BCR (7).

### Activation of Mature B Cells and Germinal Center Reaction

If mature B cells are activated through binding of an antigen to the BCR, and if T cell help is available, a T cell-dependent humoral immune response is initiated. After an initial encounter of antigen-specific B cells and cognate T cells in the T cell area of secondary lymphoid organs (e.g. lymph nodes), antigen-activated B and T lymphocytes migrate into B cell follicles and establish germinal centers (GCs). In the dark zone of these structures, the activated B cells proliferate (8). These dark zone GC B cells also activate the process of somatic hypermutation (SHM), which introduces mutations at a very high rate (10^-3^ to 10^-4^ mutations/bp per cell division (9)) into the Ig heavy and light chain V region genes. The key enzyme for this process is activation-induced cytidine deaminase (AID) (10, 11). As the mutations are largely random, most will be disadvantageous and will result in death of the respective B cells. Only B cells expressing a BCR with improved affinity will be positively selected through interactions with follicular dendritic cells and follicular T helper cells. This interaction takes place in the light zone of the GC, where the GC B cells are mostly non-proliferating. GC B cells typically undergo multiple rounds of proliferation/mutation and selection, resulting in a stepwise improvement of BCR affinity. In the course of the GC reaction, many B cells undergo class switch recombination (CSR) to change the isotype of the Ig heavy chain from IgM and IgD to IgG, IgA, or IgE (10). Also for this process, AID is an essential enzyme. Migration of the B cells within the GC is controlled by dynamic expression of the chemokine receptors CXCR4 (highly expressed on B cells in the dark zone) and CXCR5 (highly expressed on B cells in the light zone), and gradients of their ligands CXCL12 and CXCL13, respectively (12).

The transcription factor BCL6 is the master regulator of the GC B cell gene expression program (13). BCL6 activates PAX5, BACH2, and MITF, and it inhibits the plasma cell master regulators IRF4, BLIMP1 and (indirectly) XBP1 (14). Strong BCR activation leads to a shift from BCL6 dominance to upregulation of BLIMP1 (PRDM1) (14). BLIMP1 represses transcription of BCL6 and PAX5, and induces expression of IRF4 (MUM1) and XBP1, leading to differentiation of GC B cells into plasma cells. Other positively selected GC B cells differentiate into long-lived memory B cells, but the responsible transcription factor networks are less well understood (15).

### Mechanisms of Loss of Immunological Self-Tolerance

The mechanisms of BCR diversity inevitably have the side effect of generating also autoreactive BCRs (16, 17). Immature B cells with strongly autoreactive BCR are usually deleted (18), which is referred to as central tolerance. Furthermore, B cells with autoreactive BCRs can change into an anergic state (19, 20) and immature B cells with autoreactive BCRs can modify their light chain genes by new rearrangements, which is called receptor editing, and thus escape clonal deletion (21–23). Failure of the tolerance process leads to the generation of naive mature autoreactive B cells (24–26). Furthermore, peripheral self-reactive B cells receiving proliferative signals *via* MHCII/T cell receptor (TCR) and CD40/CD40L interactions can be depleted in a Fas/FasL-dependent manner (27–29). Altered pro-inflammatory, B-cell-stimulating signals such as BAFF, IL-6 or CpG or anti-inflammatory, immunosuppressive signals such as IL-10 can influence these peripheral self-tolerance checkpoints (29–31).

The presence of certain types of HLA (32) is a crucial prerequisite for most autoimmune phenomena. In addition, there are a large number of single nucleotide polymorphisms (SNPs) or mutations in susceptibility genes associated with autoreactivity, including PTPN22, CTLA4, A20, TLR7, TLR9, MYD88, CD40/CD40L, ICOS/ICOSL or genes in the BCR signaling pathway (33–39). In addition, external factors can create an inflammatory environment, reverse the segregation of certain antigens, or activate autoreactive bystander cells. In the presence of certain HLA types, immune responses against certain infectious pathogens can lead to autoreactivity *via* molecular mimicry (40, 41).

Another mechanism of loss of self-tolerance is the occurrence of alterations in self-proteins, either by somatic mutations or by atypical secondary modifications (42). The secondary occurrence of RPC1 autoantibodies and scleroderma in patients with a precancerous disease or cancer with somatically mutated *POLR3A* gene are examples (43). Besides somatically mutated neoantigens, posttranslational modifications (PTM) can characteristically lead to antigen-specific breaks of tolerance, (44) such as modified wheat gliadin in celiac disease (45), N-terminal acetylated myelin basic protein in multiple sclerosis (46), citrullinated fibrin/vimentin in rheumatoid arthritis (47, 48), phosphorylated SR proteins in systemic lupus erythematosus (49, 50), or phosphorylated enolase in pancreatic carcinoma (51–53) (**Table 1**). Not all of these autoantibodies differentiate between modified antigens and wildtype isoforms. It is assumed that PTM-specific T cells, in contrast to non-PTM-specific T cells, escape central negative selection in the thymus (59).

**Table 1:** Examples of post-translationally modified B-cell receptor (BCR) antigens in diseases other than lymphoma.

Disease	Antigen	Posttranslational Modification
Rheumatoid arthritis	fibrin/vimentin	citrullination (47, 48)
Juvenile idiopathic arthritis	DEK protein	acetylation (54)
Multiple sclerosis	myelin basic protein MOG	N-terminally acetylated (46) malondialdehyde (55)
SLE	SR proteins	phosphorylation (49, 50)
Celiac disease	Gliadin	deamidated (45) by transglutaminase
Goodpasture syndrome	collagen IV	sulfilimine bonds (56, 57)
IgA nephropathy	IgA	galactose-deficient IgA (58)
Pancreatic adenocarcinoma	Enolase	phosphorylation (51–53)

### Germinal Center Reaction and Lymphoma Genesis

The two processes modifying IgG genes in GC B cells – SHM and CSR – have not only very important roles for an efficient humoral immune response, but they also bear an inherent risk for mutations. SHM is not completely restricted to the IgV genes and can also target some non-Ig genes, including the proto-oncogene *BCL6*. This off-target SHM is particularly extensive in some types of lymphomas, including diffuse large B cell lymphoma (DLBCL), and is therefore termed aberrant SHM (60–62). Both SHM and CSR are mechanistically linked to DNA strand breaks, which is why both of them can also cause chromosomal translocations (63). Translocation of *BCL6* or *MYC* into the Ig loci are prototypical examples of such translocations mediated by misguided SHM or CSR (64). Notably, also the translocation events in GC B cells are mostly targeted to the non-expressed Ig alleles (as described earlier for V(D)J recombination-associated translocations), indicating that also at this stage of B cell differentiation, the occurrence of an oncogenic translocation does not inevitably prevent the selection for expression of a functional BCR by the lymphoma cells. Two further vulnerabilities of GC B cells for lymphoma genesis are the intrinsically high and fast proliferation activity of GC B cells, and the transient down-regulation of DNA damage responses. This allows SHM to occur without automatic induction of apoptosis (65). All these features together likely explain why about 90% of lymphomas are of B cell origin, mostly induced during a GC reaction.

Key signaling pathways frequently affected in lymphoma genesis are the following ones: the BCR- pathway with *CD79B* and/or *MYD88* mutations in the activated B cell (ABC) type of DLBCL (66), the latter also being typically involved in lymphoplasmocytic lymphoma (67), the canonical and the alternative NF-κB signaling pathway in a variety of different lymphomas including classical Hodgkin lymphoma (68–70), the NOTCH1 signaling pathway in chronic lymphocytic leukemia (CLL) (71) and a DLBCL subgroup with poor prognosis (72), the NOTCH2 signaling pathway in splenic MZL (73), as well as the JAK-STAT pathway, especially in classical Hodgkin lymphoma (74) and in primary mediastinal B-cell lymphoma (75). Furthermore, frequent mutations are described in genes encoding factors of the apoptosis signaling pathway (76) and in genes encoding for important molecules of immune surveillance (77–79).

Typically, the malignant lymphoma cells retain many characteristics of their origin counterparts, including their morphology, surface markers and gene expression profiles (7). For example, the differentiation between GC B cell-like (GCB) and ABC type of DLBCL is based on gene expression profiles (80) and immunophenotypical profiles (81). These original cell characteristics, which transformed cells can retain as established cell lines even after decades of cell culture, mostly also include the expression of the BCR. Subgroups of some lymphomas (e.g. Burkitt lymphoma (BL), primary central nervous system lymphoma (PCNSL), DLBCL, marginal zone lymphoma (MZL), MCL, and CLL) express a functional BCR, partly despite persistent AID expression with variable persistent SHM. This indicates a certain dependence or a selection advantage by BCR expression, possibly even a permanent BCR stimulation by an antigen in subgroups of the above mentioned lymphoma. In addition to the typical translocations as well as activating mutations of proto-oncogenes and inactivating mutations of tumor suppressor genes, the involvement of the BCR in lymphoma genesis was suspected early on (7, 82). The strong upregulation of the NF-κB signaling pathway in many B-cell lymphomas could also be partly explained by BCR activation.

Two principal types of BCR signaling are being distinguished. Tonic signaling is a constitutive and presumably antigen-independent signaling that is crucial for B-cell survival. In contrast, crosslinking of the BCR by direct binding to the cognate antigen induces activation of the B cell and plays an important role in humoral responses inducing B cell proliferation, AID expression, affinity maturation and differentiation. Whereas tonic signaling mainly relies on the PI3K/AKT pathway, the NF-κB pathway plays a major role in antigen-mediated active BCR signaling.

If the concept of two types of BCR signaling is applied to lymphoma, a tonic BCR stimulation pattern plays a major role in GCB-DLBCL, mainly mediated by a Y188 mutation within *CD79A*, and in a relevant subgroup of BL accompanied by mutations in *TCF3* and *ID3*, and activation of the PI3K pathway (83, 84). Active BCR signaling in lymphomas shares similarities with BCR stimulation by exogenous cognate antigens and plays an important role in ABC-DLBCL, where it is called chronic active BCR signaling (80). In ABC-DLBCL, mutations of components of the BCR pathway, including members of the CARD11/BCL10/MALT1 (CBM) complex (85, 86), and of *MYD88* (87) were frequently found. In ABC-DLBCL (88) and in CLL (89) constitutive BCR clustering is observed as it is seen in normal B cells after BCR binding of an antigen. For a particular genotypic subgroup of ABC-DLBCL with *MYD88* L265P and *CD79B* mutations, an interaction of the BCR with MYD88 was reported, mediated by TLR9 (90), which is located in the endosome and normally senses CpG DNA. This was named My-T-BCR supercomplex (91).

## Specific BCR Antigens in Lymphoproliferative Diseases

When considering BCR stimulation by antigens in lymphomas, several questions arise: is the BCR polyreactive or specific for one antigen? Are there random antigens for each individual patient with lymphoma or is there an over-representation of certain antigens? If so, are these over-representations entity-specific? And what are the possible underlying causes of the misdirected immune responses? We will highlight antigens of infectious origin as possible triggers of (mainly indolent) lymphomas. Subsequently, the involvement of autoantigens and underlying mechanisms of autoreactivity will be discussed.

### The Role of Infectious Agents in B-Cell Neoplasia

The suspected relevant BCR target antigens could be antigens of persistent or recurrent infections. The most prominent example for this is the involvement of *Helicobacter pylori* in the pathogenesis of MALT lymphoma (a subtype of MZL) of the stomach (92–95). However the BCR were found to resemble rheumatoid factor (96, 97) or in other reports were polyreactive to autoantigens including IgG and *Helicobacter* sonicate (95). This indicates a mainly indirect role of bacterial infection for triggering lymphoma growth, presumably mediated to *H. pylori*-stimulated T helper cells. Regarding the gastrointestinal tract, *Campylobacter jejuni* was also associated with the genesis of lymphoproliferative diseases in the small intestine (98). It was furthermore speculated that *Campylobacter jejuni* may also contribute to duodenal FL, as it is typically restricted to this anatomic site. Considering the usually favorable outcome, this is often managed with a watch & wait strategy. Beside gastric MALT lymphoma other entities of MZL are triggered by chronic infections. For MALT lymphoma of the ocular adnexae, a strong association with *Chlamydia psittaci* was reported in specific regions (99), and for primary cutaneous MZL, *Borrelia spec.* infections were reported to be potentially causative. Splenic MZL shows an over-representation of the IGHV1-2*04 gene, has recurrent mutations in *NOTCH2*, and in a fraction of cases its development may be triggered by hepatitis C virus (HCV) (73, 100, 101).

Successful therapeutic concepts for infection caused MZL have been demonstrated by eradication of *H. pylori* with proton pump inhibitors with or without bismuth, in combination with clarithromycin and metronidazole or amoxicillin for gastric MALT lymphomas. These drugs have been incorporated into the current therapeutic standard of ESMO/EHA for gastric MALT lymphomas regardless of stage (94, 102, 103). Furthermore, the efficacy of antibiotic eradication of *Chlamydia psittaci* for ocular adnexal MALT lymphomas by doxycycline or clarithromycine has been demonstrated (104, 105). Moreover, preliminary reports about successful treatment of *Borrelia*-spec. associated primary cutaneous MZL were published (106). Similarly, the eradication of HCV and thus elimination of viral antigens as triggers of lymphoma BCRs can lead to regression of HCV-associated splenic MZL, and this is currently recommended as first line therapy in the current ESMO/EHA guidelines (101, 103). In summary, the examples presented here highlight the potential of lymphoma regression upon anti-viral or anti-bacterial treatment. This is a strong argument for a causative role of the respective infections for sustained triggering of lymphoma growth.

**Figure 1:**
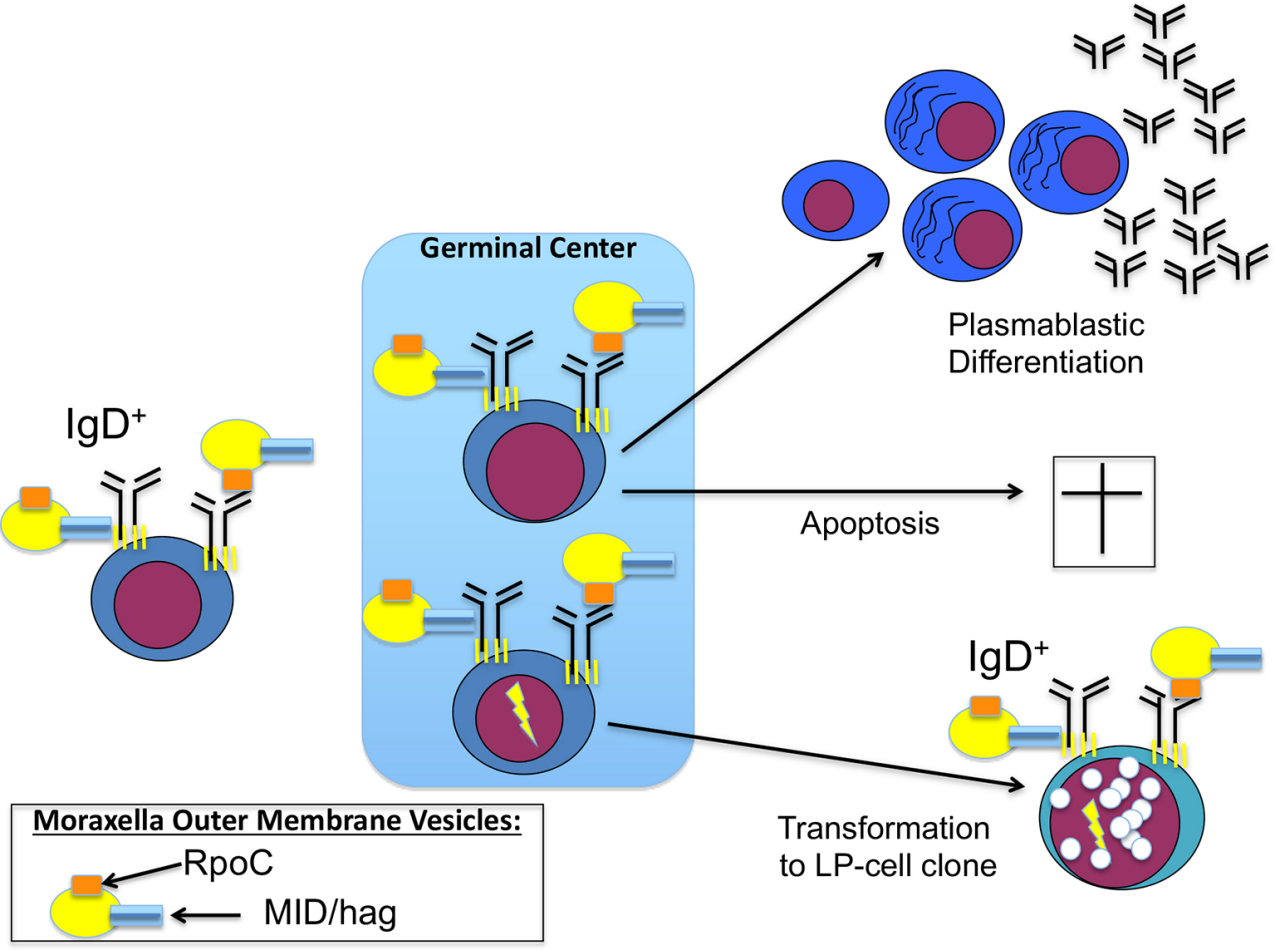
Contribution of *Moraxella catarrhalis* to IgD^+^ nodular lymphocyte predominant Hodgkin lymphoma (NLPHL) pathogenesis: costimulation of IgD-positive B cells by *Moraxella catarrhalis* RNA polymerase beta’ (RpoC) *via* the Fab fragment and MID/hag *via* the Fc fragment of the B-cell receptor (BCR). Naive IgD^+^ B cells with a BCR specific for RpoC encounter *M. catarrhalis* outer membrane vesicles. Binding of RpoC to the Fab and of MID/hag to the Fc of membrane IgD induces activation of RpoC-specific IgD^+^ B cells, which is supported by CD4^+^ T cells particularly in patients with an HLA-DRB1*04 haplotype. The persistent/recurrent presence of *M. catarrhalis* presumably induces a germinal center (GC) reaction resulting in differentiation of memory B cells and plasmablasts and production of class-switched anti-RpoC serum antibodies and apoptosis of some GC B cells due to disadvantageous mutations. Subsequently, transformation into lymphocyte predominant (LP) cells may occur, accompanied by mutations in proto-oncogenes and tumor suppressor genes, and by chromosomal translocations (e.g. *BCL6* translocations).

Using BCR expression cloning and subsequent antigen screenings, we could extend this list of infection-triggered lymphomas. We identified a specific antigen of a common bacteria as BCR antigen of nodular lymphocyte predominant Hodgkin lymphoma (NLPHL), a rare type of B-cell lymphoma, which frequently manifests at cervical lymph nodes and with regular expression of functional BCRs. This target antigen was DNA-directed RNA polymerase beta’ (RpoC) of the Gram-negative cocci *Moraxella catarrhalis* (107). *Moraxella catarrhalis* is known to co-express MID/hag, a superantigen activating IgD^+^ B cells by binding to the Fc domain of IgD. RpoC and MID/hag additively activate the BCR and the NF-κB pathways and induce proliferation of lymphocyte predominant (LP) tumor cells of NLPHL with RpoC-specific BCRs. In particular, RpoC was a frequent antigen of BCRs of IgD^+^ LP cells, whose IgHV genes had extraordinarily long complementary determining region 3 (CDR3s). Moreover, patients showed a predominance of HLA-DRB1*04/07, suggesting existence of a permissive MHC-II haplotype (107, 108). Interestingly, this haplotype is also known from autoimmunity for its association with rheumatoid arthritis as shared epitope (109). Patients had high-titer, and light-chain-restricted anti-RpoC serum-antibodies, further supporting infection of the patients by *M. catarrhalis* and mislead immune responses against this bacteria (**Figure 1**). These results suggest to conduct clinical trials examining a potential effect of antibiotic therapy for relapsed or refractory IgD^+^ NLPHL. Moreover, if no B-cell depletion was therapeutically induced in the patients, active vaccination might make sense after local therapy. For this potential strategy the target antigens, i.e. RpoC and MID/Hag of the lymphoma BCRs must not be present in the vaccine, to avoid stimulation of remaining LP cells.

Regarding aggressive lymphomas like BL, potential infectious triggers of the BCR are discussed for many years. Expression of sIg is a hallmark of all types of BL, despite *MYC*-involving translocations with one Ig gene allele as t(8;14), t(2;8) or t(8;22). However, for BL no direct BCR stimulation by an antigen has been suspected, but actually tonic BCR pathway activation amplified by mutations in *ID3* and *TCF3* genes has been reported in BL (84). CRISPR-screening identified CD79B-dependency in the BL Ramos cell line (110). For endemic BL a frequently preceding coincidence of malaria and latent EBV infection was observed, which both likely contribute to BL pathogenesis (111, 112). However, in this case EBV is not a BCR stimulating antigen, but it infects B cells and can contribute to their transformation through expression of EBV-encoded genes in latently infected B cells. For BL, in general EBV latency phase I is observed with expression of just EBNA1, so that the pathogenetic role of EBV in BL is still not fully understood (113). In sporadic BL the frequent extranodal manifestation in the appendix vermiformic and ileocoeliac junction area raised speculations about a possible infectious trigger, but a causative infectious agent has not yet been identified.

The role of infections can go far beyond direct BCR stimulation and influence lymphoma genesis in other ways. The BCR often does not seem to play a significant role in EBV-associated B-cell lymphomas, e.g. in classical Hodgkin lymphoma, functional BCR are often lost. In classical Hodgkin lymphoma, typically EBV latency II is present with expression of EBNA1, and LMP1 and LMP2a. In post-transplant lymphoproliferative disease (PTLD), EBV latency III is observed with expression of EBNA1, -2A, -3A, -3B, -3C, and LMP1 and LMP2a (113). LMP2a contains an ITAM mimicking motive (potentially) relevant for proximal BCR pathway activation, and LMP1 is a viral oncogenic mimic of CD40, recruiting among others the signaling factors TRAF2 and TRAF3, but in contrast to CD40 not TRAF3 (114–120). Besides EBV, other viruses can play important roles in lymphoma genesis by transformation of lymphocytes by latent viral infections, such as HTLV1 in adult T-cell leukemia (121) and HHV8 (122) in primary effusion lymphoma. Another important mechanism is immunosuppression by HIV attenuating control of EBV- or HHV8-infected B cells (123).

### The Role of Autoantigens in Lymphoproliferative Diseases

#### Autoantigens in Indolent Lymphoma

Endogenous immunogenic proteins could contribute to permanent growth advantages of lymphoma cells with the appropriate BCR autoreactivity by their inexhaustible supply. MZL is a CD5^-^ and CD10^-^ indolent lymphoma often accompanied by a paraprotein. Extranodal MZL is frequently associated with infectious triggers as described above. Beside recurrent mutations in *MLL2*, *NOTCH2*, *PTPRD*, and *KLF2*, nodal MZL correlates (shows) over-represented usage of *IGHV4-34* in around 30% of cases (124), which is known to be linked with autoreactivity. This autoreactivity is also demonstrated by MZL emerging from Sjögren’s disease (125).

FL is a CD5^-^ and CD10^+^ indolent lymphoma characterized by the presence of t(14;18)(q32;q21) leading to overexpression of BCL2. Regarding the BCR pathway Freda Stevenson et al. described a gain of N-glycosylation sites within the IgV genes by SHM leading to chronic activation of the BCR pathway by binding of N-hyperglycosylated BCRs to lectins in the lymphoma microenvironment (126). Subtypes of FL with a distinct manifestation and clinical course may have a different underlying biology. Here, pediatric FL, with regular cervical nodal manifestation and without *BCL2* translocation, and duodenal FL have to be mentioned. Both characteristically do not spread beyond initial local manifestations.

CLL is the most common hematological cancer in adults in the Western world and clinically shows considerable heterogeneity (127). It is characterized by a population of ≥ 5,000 clonal B cells/µl in the peripheral blood. Nodal, extranodal or splenic manifestation with < 5,000 clonal B cells/µl is called small lymphocytic lymphoma (SLL). The monoclonal tumor cells express CD5, CD23, CD200, and low levels of sIg, and lack CD10 expression. By analysis of IGHV genes of a very large number of CLL cases, it became clear that unrelated CLL patients can have highly similar if not identical BCRs (128). This phenomenon of groups of CLL with highly similar IGHV and IGHL gene rearrangements is termed BCR stereotypy. It is considered the strongest evidence that antigen selection plays an important role in the pathobiology of CLL. CLL patients whose disease belong to a specific stereotypic subset often show similar clinical and biological characteristics, including disease progression. Interestingly, for several of the stereotypic groups, autoantigen specificity of the BCR has been demonstrated (129–131). There is evidence to suggest that these BCR enable specific recognition of an (auto)antigen, which leads to increased proliferation of the malignant B-cell clone and thus plays a crucial role in the pathophysiology of CLL (132). Indeed, inhibitors of BCR signaling pathway are of great importance in clinical practice for CLL patients (133). OxLDL, Fam32a, SMCHD1, MAZ, vimentin, myosin chains, and pUL32 have been identified as (auto)antigens that can specifically bind to CLL BCRs (134–137). The mutation status of the BCR of CLL clones represents a strong prognostic marker. CLL with no or few somatic BCR mutations within their rearranged IGHV genes (“unmutated” CLL, U-CLL) experience a significantly more aggressive disease than patients with >2% mutation load (“mutated” CLL, M-CLL) (138). However, this subdivision might be more complex regarding the clinical heterogeneity of CLL. If the findings of the subsets are combined with the findings of U-CLL vs. M-CLL, there are three categories: CLLs with stereotypic BCRs (about 1/3 of the cases and mostly U-CLL), CLLs with specific IGHV genes (U- and M-CLL) and those with heterogeneous and no particular IG features (mainly M-CLL). U-CLL have polyreactive BCRs specific for autologous neoantigens (e.g. myosin chains, vimentin, oxLDL, PC9, Fam32A, SMCHD1, and MAZ) (134), while the BCRs of some M-CLL react with foreign antigens, such as yeast derived glucans (139), or autoantigens as Fc parts of rheumatoid factors (140).

There are also indications that CLL cells show antigen-independent, cell-autonomous signaling (141, 142); a behavior that has not been shown in normal B cells and other B-cell malignancies. This cell-autonomous signaling is based on the recognition and self-association of the BCR of CLL cells to itself, the ultimate autoantigen. Various CLL-derived BCR bind to specific, different epitopes of themselves and thus initiate intracellular signal transduction. The avidity of BCR self-recognition seems to have an influence on the course and severity of the disease. In summary, for CLL different models and ideas exist for the significance of the BCR in CLL. It is likely that all models of BCR reactivity have their justification, whereby, depending on the situation, one or the other mechanism may be more important. All models emphasize the importance of BCR antigen recognition in conjunction with BCR auto-stimulation in addition to genetic lesions in the pathogenesis of CLL. However, it should not be forgotten that additional effects with an influence on the pathogenesis have to be considered, such as the existence of specific effector functions for IgM and IgD. Nevertheless, the use of inhibitors of the BCR signaling pathway (e.g. BTK inhibitors) and thus the proliferation of CLL cells has significantly improved the therapeutic options and led to permanent remissions, even in high-risk and refractory CLL patients.

Hairy cell leukemia (HCL) is a rare indolent lymphoma typically affecting middle-aged to old males. Beside its name-giving feature of protruding villi on the surface of the leukemic cells, it has an immunophenotype characterized by expression of CD103, CD11c, CD22, and CD123, and lack of CD5 and CD10 expression. Expression of CD25 distinguishes a classical and a variant form of HCL (143). Classic HCL always carry *BRAF* mutations (144). HCL cells express a BCR and the IGHV genes IGHV3-21, IGHV3-30, and IGHV3-33 are overrepresented. HCL is frequently associated with Igλ light chains (145, 146). Variant HCL lack *BRAF* mutations, and often use the IGHV4-34 gene.

MCL is a rare B-cell neoplasia, which accounts for about 6-8% of all Non-Hodgkin lymphoma (127). Male, elderly patients are over-represented, and extra-lymphatic manifestations are common (147, 148). MCL cells typically show a CD5^+^, CD23^-^, CD200^-^ immunophenotype with strong expression of CyclinD1, due to translocation of the *CCND1* gene into the IgH locus (t(11;14)(q13;q32)) (149). Regarding its IGHV mutational and DNA methylation status, MCL can be distinguished into pre- and post-GC-derived cases (150). Over-representation of specific IGHV gene groups and stereotypic rearrangements has been described similar to CLL, but with a lower frequency (151–153). In accordance with this, a strong BCR and NF-κB pathway activation in MCL was reported (154), and antigen-induced activation was stronger compared to other B-cell neoplasia (155). Pharmacological targeting of the BCR pathway by inhibition of PI3K or BTK is established for relapsed/refractory MCL (156–159). Recently, we identified human LDL receptor-related protein associated-protein 1 (LRPAP1) as frequent autoantigen of recombinant BCRs in MCL cases (8/21) and two of seven MCL cell lines (MAVER1 and Z138) (160). LRPAP1 consists of 357 amino acids and has a molecular weight of 39 kDa. LRPAP1 functions as antagonist and chaperon of the family of LDL-receptors and it takes part in Megalin/Cubilin endocytosis (161, 162). Immunization of rats with LRPAP1 results in Heymann-Nephritis (163, 164).

#### Autoantigens in Aggressive Lymphomas

**Figure 2:**
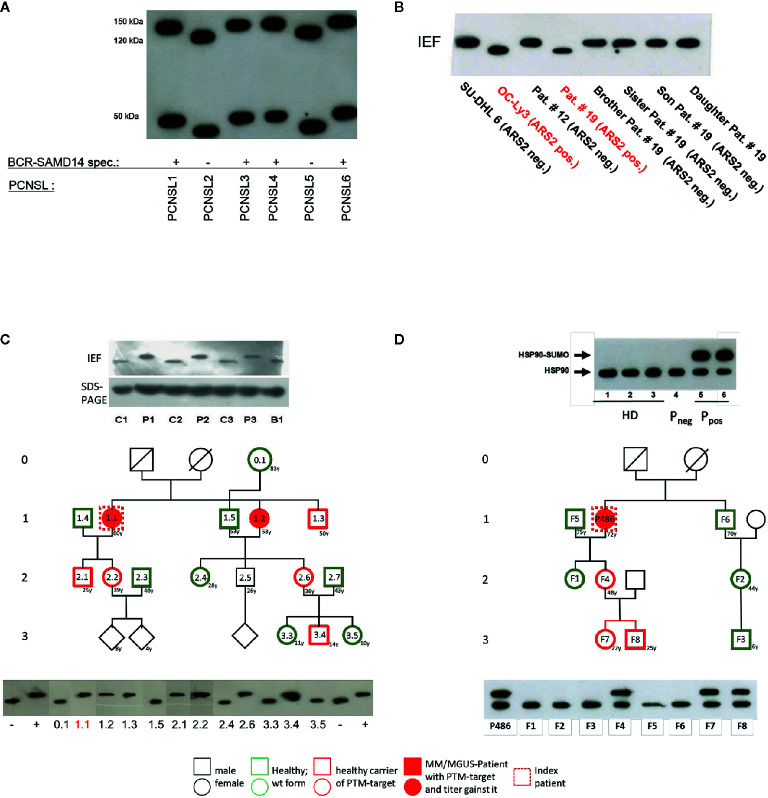
Examples of posttranslational modification of lymphoma B-cell receptor (BCR) target antigens: **(A)** Representative Western blot of hyper-N-glycosylated Neurabin-I and SAMD14 in patients with PCNSL first reported by Thurner et al (173). Patients with primary central nervous system lymphoma (PCNSL) and SAMD14/Neurabin-I reactive lymphoma BCRs had exclusively hyper-N-glycosylated isoforms of both antigens. **(B)** Representative isoelectric focusing (IEF) of hypophosphorylated Arsenite resistance protein 2 (ARS2) in diffuse large B cell lymphoma (DLBCL) first reported by Thurner et al (174). DLBCL cell lines and peripheral blood lysates of DLBCL of patients and family members. ARS2 was found to be hypo-phosphorylated in a cell line and a patient, but this phenotype of an atypical posttranslational modifications (PTM) was not inherited in a Mendelian manner. **(C)** Representative analysis of hyperphosphorylated SLP2 (paratarg-7), which was first reported by Preuss et al (171). Hyperphosphorylated SLP2 was detected by IEF and not by SDS-PAGE. The hyperphosphorylation introduces an additional phosphate group in the molecule leading to a different isoelectric point of the protein; the increase in molecular weight is too small to be detected by SDS PAGE. Shown are immunoblots incubated with anti-human-STOML2 (paratarg-7). P1-3: MM/monoclonal gammopathy of undetermined significance (MGUS) patients with immunoreactivity against paratarg-7; C1-3: MM/MGUS patients without SLP2 (paratarg-7) immunoreactivity; B1: healthy blood donor. Inheritance: SLP2- phosphorylation state in patient families (example). The family of a relevant patient was analyzed for its SLP2 phosphorylation state by IEF. Carriers of hyperphosphorylated SLP2 (patient and persons of risk) were indicated in red. **(D)** Representative SDS PAGE of sumoylated HSP90 first reported by Preuss et al (175). Sumoylation of HSP90 does not change the isoelectric point of the molecule but leads to an increase in molecular weight which is detected by SDS PAGE. HD: healthy donor; P_neg_: MM/MGUS patient without immunoreactivity against HSP90-SUMO; P_pos_: MM/MGUS patients with immunoreactivity against HSP90-SUMO.Inheritance: HSP90 sumoylation state in patient family (example). The family of a relevant patient was analyzed for HSP90-SUMO by SDS PAGE. Carriers of HSP90-SUMO (patient and persons of risk) are indicated in red.

Regarding autoantigenic targets of BCRs of aggressive lymphomas, several examples exist. DLBCL is the most common aggressive B-cell Non-Hodgkin lymphoma. According to the WHO classification, DLBCL can be classified based on gene expression profiling into ABC–like type, GCB–like type and primary mediastinal B-cell lymphoma (80, 165). In contrast to relatively well studied genetic or epigenetic pathway alterations, little is known about specific external stimuli of distinct subgroups of DLBCL (166, 167). In particular, DLBCL of the ABC-type or the recently specified MCD-type or cluster 5 harbor recurrent mutations in *MYD88* and *CD79B* with dependency on constitutive BCR signaling (72, 88, 168, 169). For systemic DLBCL a cis and trans stimulation of the BCR by a so far non-characterized autoantigen was reported for the HBL1 cell line. Moreover, an anti-idiotype reactivity of the BCR of the TMD8 cell line against an epitope within its own FR2 (V^37^R^38^) was described, and for the U2932 and OCI-LY10 cell lines BCR reactivity against apoptotic cell debris was reported (169–172). Recently, Arsenite resistance protein 2 (ARS2) was identified as the BCR target of ABC-DLBCL. Compared to controls, ARS2 was hypo-phosphorylated exclusively in cases and cell lines with ARS2-specific BCRs (**Figure 2** and **Table 2**). In a validation cohort, hypo-phosphorylated ARS2 was found in 8/31 ABC-DLBCL, but only 1/20 GBC-DLBCL. Incubation with ARS2 induced BCR-pathway activation and increased proliferation, while an ARS2/ETA’ toxin conjugate induced killing of cell lines with ARS2-reactive BCRs.

**Table 2:** Post-translationally modified B-Cell Receptor (BCR) antigens in lymphoma.

Disease	Antigen	Posttranslational Modification
CLL	LDL	oxidization (176)
PCNSL	SAMD14/neurabin-I	N-glycosylation (173)
DLBCL	ARS2	Hypophosphorylation
BL	HSP40, Bystin	sumoylation and acetylation
LPL/MM	SLP2, ATG HSP90 LGL1, sapC	phosphorylation (177–179) sumoylation (175) deficiency glucocerebrosidase (180)

PCNSL represents a specific extranodal subtype of DLBCL with molecular similarities to systemic DLBCL of MCD or C5 type with frequent mutations in *MYD88* and *CD79* (195, 202). PCNSL show strong over-representation of IGVH4-34, and poly-reactivity against a plethora of antigens was reported (196, 203). In addition, sterile a-motif domain containing protein 14 (SAMD14) and neural tissue-specific F-actin binding protein I (neurabin-I) with a homologous SAM domain were identified as specific auto-antigenic targets of recombinant BCRs of PCNSL and SAMD14/neurabin-I specific autoantibodies were detected in sera and cerebrospinal fluid of patients. In the respective cases, SAMD14 and neurabin-I were atypically hyper-N-glycosylated (SAMD14 at ASN339 and neurabin-I at ASN1277), explaining their auto-immunogenicity (**Figure 2**) (173). Primary intraocular lymphoma (PIOL) is biologically closely related to PCNSL and can progress after a various period of time into PCNSL (198), but it is not clear whether SAMD14/neurabin-I are altered as well in this subgroup, or if a different antigenic trigger exists.

Although tonic BCR activation is characteristic for BL (84), preliminary results suggest the involvement of post-translationally modified specific autoantigens that contribute to pathogenesis in at least a subgroup of sporadic EBV-negative BL (191).

#### Autoantigens in Plasma Cell Dyscrasia

Multiple myeloma (MM) accounts for 1% of all malignancies, and for over 10% of hematological malignancies. The disease is characterized by neoplastic proliferation of a single plasma cell clone producing a large amount of a monoclonal antibody termed paraprotein, M-protein or M-component (204). Malignant gammopathies are often preceded by monoclonal gammopathy of undetermined significance (MGUS), a benign disorder with a strikingly elevated monoclonal Ig level in individuals lacking evidence of MM or other lymphoproliferative malignancies. Long-term follow-up of patients with MGUS reveals a 1% to 3% annual risk of developing MM or, to a lesser extent, other lymphoproliferative malignancies (204). In MGUS and plasma cell dyscrasia hyperphosphorylated SLP2 and sumoylated HSP90 were found to be the targets of paraproteins (171, 175) both in MM and lymphoplasmocytic lymphoma (LPL) (**Tables 2** and **3**). These “paratargets” with their atypical PTMs were found with different frequencies in different ethnics (205). Of interest, PTMs for both antigens had an autosomal dominant pattern of inheritance (**Figure 2**), and pedigrees with family members as carriers and affected with MGUS or plasma cell dyscrasia were described for both hyperphosphorylated SLP2 and sumoylated HSP90 (175, 179). Interestingly, SLP2-reactive paraproteins do not differentiate between the normally phosphorylated SLP2 and the hyperphosphorylated SLP2 isoform in contrast to HSP90-reactive paraprotein, which is specific for the sumoylated isoform.

**Table 3:** B cell lymphoma and B-cell receptor (BCR) antigens.

B-cell neoplasia	Expression of sIg	Indications for chronic BCR stimulation by an antigen or alternative BCR pathway activation
CLL	Yes, dim	subsets with stereotypic CDR3 (181) specific autoantigens for individual subsets (129, 134, 176, 182) specific microbial antigens (139) concept of autonomous, antigen-independent BCR signaling mediated by anti-framework region reactivity (142) clinical effectivity of BCR pathway inhibition (133)
MCL	Yes	subgroup with stereotypic CDR3s (152) subgroup with BCR-reactivity and autoantibodies against LRPAP1 (183) reactivity against protein A of *Staphylococcus aureus* (184) clinical effectivity of BCR pathway inhibition (156, 159)
FL	Yes	gains of N-glycosylation sites in BCR yield in binding to lectins (126) pediatric FL: cervical manifestation, speculation about infectious trigger duodenal FL: speculations about infectious trigger
HCL	Yes	classic HCL hints for affinity maturation (145, 185) variant HCL regularly *IGHV4-34*
MZL	Yes	splenic MZL: strong association with HCV (100, 101) MALT-lymphoma of the stomach: strong association with *H. pylori* (94, 95) MALT-lymphoma of ocular adnexes: reported association with *Chlamydia psittaci* (99) MALT-lymphoma of salivary glands after Sjögren’s syndrome: autoreactive BCR (125) effectivity of BCR-pathway inhibition (186)
cHL	No	destructive IgV gene mutations in 25% of cases (187, 188) *ITAM-signal of EBV-encoded LMP2a mimicking activated BCR (117)
NLPHL	Yes	reported predominance of Igκ-light chains (189) IgD^+^ subgroup with cervical manifestation (190) *Moraxella catarrhalis* RpoC as antigen of IgD^+^ LP-cells with extraordinary long CDR3s (107) However, clinical trials with BTK-inhibition in r/r NLPHL failed
BL	Yes	concept of tonic BCR activation by mutation in *ID3* and *TCF3 (* 83) suspected stimulation by *Plasmodium falciparum* of EBV-infected centroblasts in endemic BL (111, 112) reports of modified autoantigens in sporadic BL (191)
DLBCL	in subgroups Yes	ABC-type activating mutations in *CD79B* und *MYD88* (66, 87) of MCD type, cluster 5 or ABC-type reported autoreactivity of OCI-Ly10, U2932 lines (169), reactivity against FR2 of TDM8 line, cis and trans stimulation by an autoantigen for HBL1, reactivity of CDR3 of TDM8 against FR2 (V (37)R (38)) of TDM8 (169) ARS2 identified as frequent target antigen of ABC-type DLBCL. ARS2 hypophosphorylated in these cases. effectivity of BCR-pathway inhibitors (192, 193) PCNSL overrepresentation of auto-reactivity associated *IGHV4-34 (* 194) activating mutations in *CD79B* und *MYD88 (* 195) reported poly-reactivity of BCR (196) SAMD14/neurabin-I identified as target of BCRs SAMD14/neurabin-I hyper-N-glycosylated in these patients (173) effectivity of BCR-pathway inhibitors (197, 197) PIOL shares biologic characteristics with and frequently progresses to PCNSL and shares overrepresentation of IGHV4-34 (198) PTL frequently shares activating mutations in *CD79B* und *MYD88*, with other aggressive lymphomas of immunologically privileged sites
PMBCL	No	probably independent of BCR (199)
LPL	Yes	clinical effectivity of BCR pathway inhibition (200, 201) post-translationally modified SLP2 and HSP90 as specific antigens (paratargets) of IgM paraproteins (171, 175, 180)
MM	only secreting, no sIg	posttranslationally modified SLP2, HSP90, sapC as specific antigens (paratargets) of paraproteins (171, 175, 180)

The question as to why the respective paraprotein antigen is present in post-translationally modified form in this group of people and to what extent this influences the development and progress of the disease remains unsolved. It is remarkable that these post-translationally modified antigens were detected almost exclusively in MM/MGUS patients and their blood relatives as well as in approx. 1-2% of the healthy population; in all other examined subjects (patients and healthy persons) the antigen is present in wild-type form, i.e. unchanged and does not induce an immune response. Nair et al. had different findings and described glycosphingosine as a frequent antigenic target structure of paraproteins in sporadic MGUS and MM as well as in monoclonal gammopathies associated with Gaucher disease (180, 206). Data from our laboratory rather suggest post-translationally modified saposin C as a paraprotein target structure in Gaucher-associated MM/MGUS (207).

#### Role of T-Helper Cell Co-Stimulation in Lymphoproliferative B-Cell-Diseases

Sequence and structure of the BCR antigens of malignant plasma cells found to date, whether post-translationally modified or not, indicated the need for the involvement of T-helper cells for stimulation at the beginning and during the course of pathogenesis. Furthermore, several studies provided evidence for a causal relationship between MGUS/MM and chronic antigenic stimulation (208). In addition, when SLP2 is used as a model antigen in MM, the patient’s paraprotein binds to both the wild type SLP2 and the actually immunogenic post-translationally hyper-phosphorylated variant of SLP2. Thus, SLP2-specific B cells cannot be the initiators of the postulated chronic stimulation or pathogenesis.

**Figure 3:**
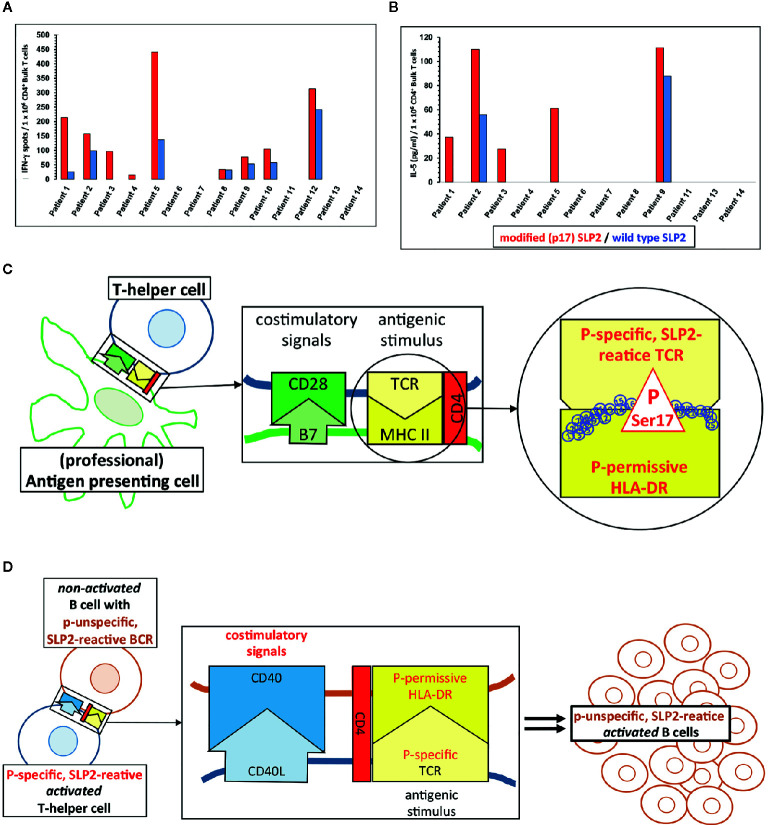
Paratarg-7-specific T-helper cells in myeloma/monoclonal gammopathy of undetermined significance (MGUS) patients with a SLP2-specific paraprotein as a new type of epitope spreading. **(A)** Representative IFN-g ELISPOTs first reported by Neumann et al. showed by *in vitro* stimulation for SLP2(paratarg-7)-specific TH1 cells in 9/14 patients specific responses to the antigen. 8/9 patients had a significant (p << 0.05.) stronger response against the phosphorylated variant of the SLP2-peptides used for stimulation (red columns) compared to the peptides derived from the non-phosphorylated wild type (blue columns) (209). **(B)** T-helper cells from 5/12 patients showed a significant stronger TH2 response against the modified peptides compared to the wild type peptides. Again, these are the results of *in vitro* stimulation of myeloma/MGUS patients’ T-helper cells with a paratarg-7/SLP2-specific paraprotein using overlapping 15 amino acids long peptides covering the first 30 amino acids of the SLP2 sequence. Wild-type peptides and peptides with a phosphorylated Ser17 of the posttranslational modifications (PTM) variant were used. Subsequently, the culture supernatant was analyzed by ELISA for the TH2 cytokine IL-5 (209). **(C)** T-helper cells with a T-cell receptor (TCR) specific for Ser17-phosphorylated version of SLP2 are primed by antigen-presenting cells, equipped with the corresponding permissive MHC-II molecules offering all necessary costimulatory signals for full maturation. **(D)** Subsequently, T-helper cells with these properties (phosphospecific SLP2-reactive) can stimulate all B cells presenting a phosphorylated Ser17 epitope. The specificity of the receptor of these B cells for the hyperphosphorylated isoform of the antigen is not important. Thus, B cells are also stimulated whose BCRs bind the unmodified wild type of SLP2. This form of epitope spreading comprises the same amino acid of the antigen, but with the difference of posttranslational modification. Therefore, this type of spreading is vertically oriented. For some other posttranslationally modified antigens, the lymphoma BCRs are specific for modified isoform/variant depending on the PTM, i.e. HSP90-SUMO.

*In vitro* stimulation of CD4^+^ T-helper cells of MGUS/MM patients with a paratarg-7-specific paraprotein induced distinct paratarg-7-specific responses: 65% of these patients had a paratarg-7-specific TH1 response. 89% of these TH1 cells specifically recognized the modified hyperphosphorylated SLP2. 42% of the stimulated patients also had modification-specific TH2 cells (**Figures 3A, B**). Hence, with the T-helper cells the contribution of the adaptive immune system was found, which specifically recognize the post-translational modification and thus are at the beginning of chronic stimulation. Further characterization of the hyperphosphorylated SLP2-specific T-helper cells showed that there are (at least) six HLA-DR subtypes, named “permissive”, that can present phosphorylated peptide epitopes to the T helper cells for stimulation. Compared to a healthy reference population, patients with SLP2-specific MM express these six permissive DR subtypes significantly more frequently. Thus, expression of a hyperphosphorylated-SLP2-permissive DR subtype is, besides posttranslational modifications, the second important prerequisite for the development of this disease (209).

The interaction of these modification-specific T-helper cells from patients with non-modification-specific paraprotein and their corresponding B cells is a new type of epitope spreading. In contrast to classical epitope spreading, which extends horizontally across the amino acid sequence of an antigen, this is a vertically spread modification of the same amino acid.

Physiologically, however, both phenomena are based on the same fact that the epitope of an antigen that is specifically bound by the BCR of a B cell does not have to be the same epitope that the B cell presents to the T-helper cells in its MHC-II molecules after antigen processing. When a T-helper cell finds its antigenic epitope in the appropriate MHC-II context on the B cell, it provides the help necessary for the maturation of this B cell. Applied to the situation with SLP2, this means that even those SLP2-specific B cells whose BCR does not differentiate between the modified version of this antigen phosphorylated on serine17 and the non-phosphorylated wild-type, can be stimulated by modification-specific T-helper cells (**Figures 3C, D**).

Since all posttranslational paratarg modifications found to date (including SLP2) are always consistently expressed in all cells of the organism, the B cells of the respective patients with a paratarg-specific BCR as well as all other antigen-presenting cells (e.g. macrophages or DC) can present only the modified variant to their T-helper cells. This can be seen by the fact that, except in the TH1 response of patient 8, the induced SLP2-specific T-cell responses were significantly modification-specific after *in vitro* stimulation of the T-helper cells (**Figures 3A, B**).

### B-Cell Lymphoma Without a Role of BCR Antigens

**Figure 4:**
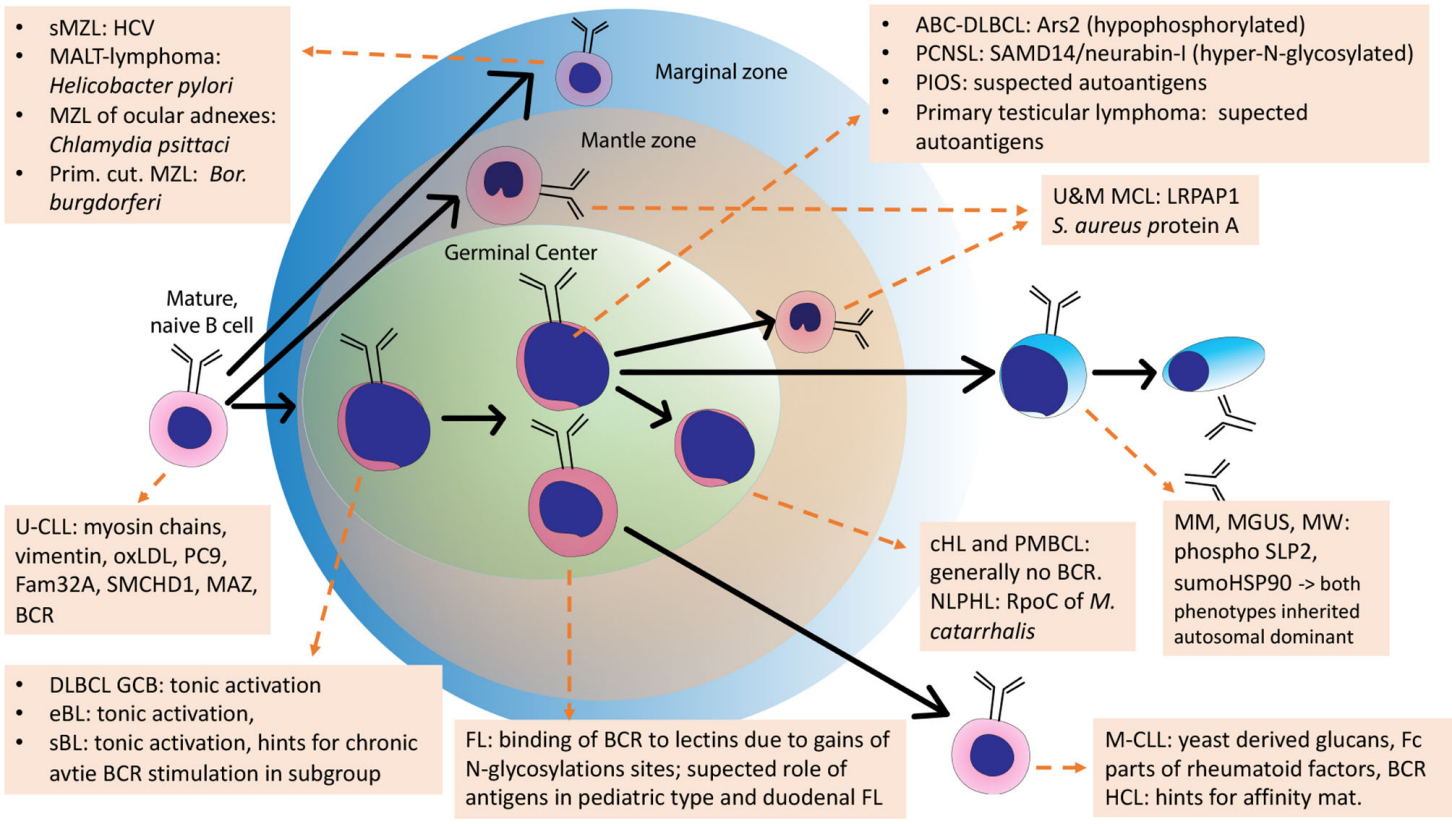
Schematic overview of the development of normal B cells and their malignant counterparts. Arrows: Normal B-cell differentiation; dotted arrows: normal B-cells as cell of origins of specific lymphomas. This scheme is an adaption of the scheme from Küppers et al., 2005, with added B-cell receptor (BCR) antigens identified in the meantime.

For some other lymphoma entities, stimulation of the BCR by antigens is likely not important. These are for example classical Hodgkin lymphoma, whose malignant Reed-Sternberg und Hodgkin cells have lost their B-cell-phenotype including functional BCR genes (187), PTLD with crippled BCR genes (210), or primary mediastinal B cell lymphoma (211, 212), which usually does not express sIg either (**Figure 4**).

### Suspected Role of Specific Antigens in T Cell Neoplasia

The search for TCR antigens is much more complicated as it requires screening of peptides presented on correct HLAs. This is probably the main reason why no TCR target antigens of PTCL have been found. For certain peripheral T cell lymphomas (PTCL) there is evidence for a potential role of antigen stimulation in their pathogenesis. One example is angioimmunoblastic T cell lymphoma, in which clonal B cell populations and paraproteins are often also found. It is unclear if the paraproteins in AITL might be a simple epiphenomenon of accompanying EBV^+^ B-cell-clones, or if they target the same antigen as the TCRs of the T follicular helper cells of origin of AITL. Another example is enteropathy-associated T cell lymphoma, which is rare and typically occurs after a long period of celiac disease. Breast implant-associated anaplastic T cell lymphoma usually shows a significantly more favorable course than ALK-negative ALCL and the role of antigen stimulation is partly shown by remission after explanation of the breast implants alone. However, a TCR-reactivity against components of breast implants has not been shown.

## Lymphoma BCR and Therapeutic Implications

### Inhibition of the BCR Pathway

In the treatment of B-cell lymphomas and leukemias and increasingly in the treatment of autoimmune diseases, the inhibition of the main signaling pathway of B cells, the BCR pathway, plays a crucial role. SYK inhibitors make pathogenetically sense because the SYK kinase lies relatively far upstream in this pathway. SYK inhibitors showed *in vitro* and *in vivo* activity against B-cell lymphomas and various other hematological neoplasias. However, SYK inhibitors have not yet been able to gain clinical importance in the treatment of B-cell lymphomas (213). Interestingly, SYK inhibitors were investigated for autoimmune diseases with major B-cell involvement, including rheumatoid arthritis (214), and the first FDA approval of a SYK inhibitor, fostamatinib was granted for treatment of immune thrombocytopenia (ITP) (215). The BTK inhibitor ibrutinib initially represented a new standard in the therapy of r/r CLL showing even enduring responses in CLL with *TP53* mutations or del17p (133), and later it was shown that even in the therapy of CLL with mutated IGHV status the results were superior to the previous gold-standard immunochemotherapy concepts (216). In these cases, the accumulation of cancer cells is usually slowed down, but no complete remission in terms of negative minimally residual disease (MRD) is achieved. The C481S mutation in BTK and various PLCg2 mutations as well as mutations in the PI3K signaling pathway have been identified as resistance mechanisms (217, 218). Further indications for which ibrutinib is approved are LPL in combination with rituximab (201) and r/r MCL (156), for which the combination with the BCL2 inhibitor venetoclax was particularly impressive (159). In aggressive lymphomas, BTK inhibitors appear to be of particular benefit in lymphomas with activating mutations in *MYD88* and *CD79B* (91). In PCNSL, BTK inhibitors have been used as monotherapy (197). Unfortunately, in combination with immunochemotherapy, increased mold infections were observed (219). In a DLBCL first line trial, the combination of ibrutinib with immunochemotherapy led to increased toxicity, so that immunochemotherapy could often not be completed in a relevant proportion of patients, but a subgroup analysis showed a significant improvement of overall survival for younger patients (193). Acalabrutinib is a second generation BTK inhibitor, which is also approved in CLL and shows a different spectrum of side effects as compared to ibrutinib (220–222). In contrast to ibrutinib and acalabrutinib, the non-covalent BTK inhibitors Loxo-305, Vecabrutinib and ARQ 531 do not require the presence of the C481 wild type configuration, but may also be active in C481S BTK mutated disease (223–225). Similar to SYK inhibitors, BTK inhibitors are also being investigated in autoimmune diseases and some are approved for this purpose as for ITP, multiple sclerosis or graft-versus-host disease (GVHD) (226–228). A further prominent target for inhibition is PI3K with idelalisib, copanlisib and duvelisib approved for r/r CLL/SLL and FL, the first inhibits selectively PI3kdelta, the latter inhibits PI3Kalpha and PI3Kdelta and the last PI3Kdelta and PI3Kgamma (229–231). However, the use of idelalisib was decreased due to toxicity problems, mainly related to autoimmune phenomena such as pneumonitis and colitis (232). Further potential targets for inhibition are Lyn and the components of the CBM complex, i.e. CARD11, BCL10, and MALT1 (233).

It is presently unclear why some types of lymphomas respond well to BCR inhibiting treatment and others not. Perhaps, in non-responding lymphomas, BCR pathway activity is less essential for lymphoma cell survival and proliferation, or this pathway is activated by genetic and/or epigenetic mechanisms further downstream, so that upstream inhibition of BTK does not cause a downregulation of this pathway, an example is r/r FL lymphoma with mutation in CARD11 showing poor response to ibrutinib (234).

### Attenuation of BCR Activation by Eradication of Antigenic Trigger

A possible therapeutic strategy for antigen-driven lymphomas is to remove the antigenic trigger. This can be possible for infectious antigenic triggers as typically in MZL by antibiotics or antiviral therapy (101, 103). A similar approach of antibiotic eradication could be investigated for *Moraxella spec.*-reactive NLPHL in clinical trials for patients with relapsed/refractory IgD^+^ NLPHL, or a consolidating vaccination against *Moraxella catarrhalis* in early stage NLPHL patients who have only been treated locally and have not received therapeutic B-cell depletion. Of course, this vaccination must not contain the antigenic triggers themselves.

### Attenuation of Stimulation by Modulation of Immunogenic PTMs

For lymphoplasmocytic lymphoma, for ABC-DLBCL, and for PCNSL, the search for specific substances to modulate the immunogenic PTMs of hyper-phosphorylated SLP2 and sumoylated HSP90 in LPL, of N-hyperglycosylated SAMD14/neurabin-I in PCNSL and of hypophosphorylated ARS2 in DLBCL would be useful. The aim would be to reverse the PTMs as permanently as possible and thus weaken the immunogenic stimulus. These substances could, for example, be investigated in secondary prophylaxis.

### Targeting Lymphoma-Cells by Their BCR-Reactivity

**Figure 5:**
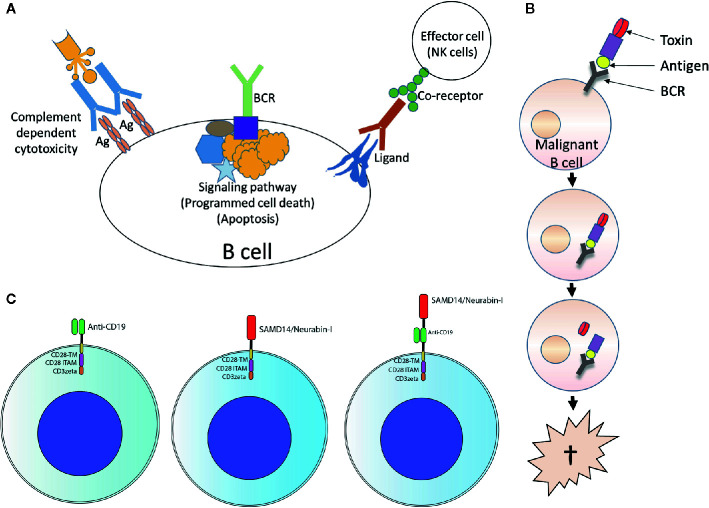
Usage of lymphoma B-cell receptor (BCR) antigens for targeting lymphoma. **(A)** Forward targeting: Classical antibody therapy cannot differentiate between malignant and benign B cells. mAbs against B cell surface antigens such as CD20 bind to their target present on all B cells and activate complement, antibody-dependent cell-mediated cytotoxicity or direct cell death. The same is also true for antibodies against receptors with immunomodulatory functions. Independently of this, tumor cell death could also be induced antibody-independently by drugs that interfere with critical signaling pathways (such as ibrutinib, which interferes with BTK, a step in the BCR signaling pathway). Ag, antigen. **(B)** Reverse targeting: The BAR (BCR antigen for reverse targeting) concept is based on the highly specific interaction of a BCR found exclusively on malignant B cells with its highly specific target antigen; benign B cells do not possess this BCR. Synthetic conjugates of BCR antigen with a toxin (BAR toxin) bind exclusively to the malignant cells, are internalized and release the toxin that kills the cell. **(C)** CARs: Conventional CAR with CD19 scFv/CD28 4-1BB CD3ζ CAR backbone (left); the anti CD19 scFv was exchanged by the frequent BCR antigen of MCL resulting in the construct SAMD14/neurabin-I/CD28 4-1BB CD3ζ CAR backbone (middle) or combined with anti CD19 scFv (right).

The specific BCR antigens identified so far could possibly be used as basis for therapeutic approaches using retrograde BCR targeting - which has been proposed as the BAR (BCR antigen for reverse targeting) concept. This approach has parallels of targeting lymphoma BCRs by anti-idiotypes (235) and exploits the entity-specific BCR reactivity of lymphoma clones. Possibilities would be, for example, immunotoxins consisting of fusion proteins with the epitope region of the target antigen coupled to a toxin or an enhancer of the immune response (236–238), or T or NK cells with chimeric antigen receptors with the epitope region of the target antigens as extracellular capture domain (**Figure 5**) (239). This approach was demonstrated *in vitro* using immunotoxins consisting of the epitope of the respective BCR target antigen and a shortened form of *Pseudomonas aeruginosa* exotoxin A (ETA’). The role of pre-existing serum autoantibodies against the corresponding target antigens is critical here. After infusion of immunotoxins, consisting of epitope region and toxin, deposits of toxic immune complexes could develop. When using chimeric antigen receptor (CAR) T cells with an epitope region of the lymphoma BCR target antigen as part of the CAR ectodomain, the antibodies could possibly cause strong stimulation with cytokine release syndrome. *In vivo*, however, such CAR T-cell constructs appear to function despite the presence of autoantibodies (239). A standard anti-CD19-scFv/CD28/CD3ζ second-generation CAR construct might be used as a basis. The target antigen epitopes are combined or replace the anti CD19 scFv.

## Author Contributions

LT wrote the manuscript. K-DP wrote the section on plasma cell diseases, FN on the role of Th cells, SH on NLPHL, and MB and K-DP on reverse targeting. RK and SS revised the manuscript and contributed significant and very important additional information. MH revised the manuscript and contributed information concerning effector cells and therapeutic mechanisms. All authors contributed to the article and approved the submitted version.

## Funding

We thank the following funding organizations for supporting lymphoma research of the authors relevant for this review: HOMFOR grants to LT and MB, Deutsche Forschungsgemeinschaft (grants KU1315/9-2 and KU1315/14-1 to RK), Deutsche Krebshilfe (grant 70112112 to RK), Wilhelm Sander Foundation (grant 2019.056.1 to LT, K-DP, FN, and SH and grant 2018.101.1 to RK), Deutsche José Carreras Leukämie Stiftung (grant 02-R/2020 to RK), the Hairy Cell Leukemia Foundation (to RK). 

## Conflict of Interest

The authors declare that the research was conducted in the absence of any commercial or financial relationships that could be construed as a potential conflict of interest.
